# Retraction Note: Noninvasive thrombectomy of graft by nano-magnetic ablating particles

**DOI:** 10.1038/s41598-022-18312-7

**Published:** 2022-08-17

**Authors:** Abbas Moghanizadeh, Fakhreddin Ashrafizadeh, Jaleh Varshosaz, Mahshid Kharaziha, Antoine Ferreira

**Affiliations:** 1 Department of Materials Engineering, Isfahan University of Technology, Isfahan, 84156-83111 Iran; 2 Department of Pharmaceutics, School of Pharmacy and Pharmaceutical Sciences, Isfahan University of Medical Sciences, Isfahan, Iran; 3 INSA Centre Val de Loire, Université d’Orléans, PRISME EA4229, Bourges, France

Retraction of: *Scientific Reports* 10.1038/s41598-021-86291-2, published online 26 March 2021

The Editors have retracted this Article.

After publication of this Article it was brought to the Editors' attention that some of the data appear to overlap with those in another article from these authors^1^. Specifically:

- the TEM image of zinc ferrite in Figure 3 appear to be identical with the TEM image of Fe_3_O_4_ nanoparticles in Figure 1 in^1^;
- VSM data for zinc ferrite in Figure 4B appear to be identical with the Fe_3_O_4_ VSM data in Figure 2B in^1^.

The Authors are unable to provide the raw data underlying these experiments; the Editors therefore no longer have confidence in the results presented.

Antoine Ferreira agrees with the retraction and its wording. Fakhreddin Ashrafizadeh, Jaleh Varshosaz and Mahshid Kharaziha disagree with the retraction. Abbas Moghanizadeh has not explicitly stated whether they agree with this retraction.
